# Multi‐environment evaluation of raffinose‐family oligosaccharide content in diverse dry bean varieties and their reduction upon canning

**DOI:** 10.1002/jsfa.70303

**Published:** 2025-11-12

**Authors:** Brendan M. O'Leary, Vinti Kumari, Todd Reid, Parthiba Balasubramanian

**Affiliations:** ^1^ Saskatoon Research and Development Centre Agriculture and Agri‐Food Canada Saskatoon SK Canada; ^2^ Department of Biology University of Saskatchewan Saskatoon SK Canada; ^3^ Lethbridge Research and Development Centre Agriculture and Agri‐Food Canada Lethbridge AB Canada

**Keywords:** abiotic stress, canning, carbohydrates, dry beans, galactose oligosaccharides, Raffinose family oligosaccharides

## Abstract

**BACKGROUND:**

The substantial amount of raffinose family oligosaccharide (RFO) in dry beans causes flatulence and digestive discomfort, limiting the appeal of dry bean food products. Substantial variation exists in RFO content among dry bean varieties, and various food processing steps such as soaking and canning are known to reduce RFO levels. However, little information exists concerning environmental effects upon RFO accumulation in field‐grown dry beans, or varietal differences in RFO reduction upon downstream processing of whole seeds.

**RESULTS:**

This study surveyed a multi‐environment trial of diverse dry bean varieties grown under irrigation to investigate sources of RFO variation. Controlled heat treatment experiments were conducted to evaluate the effect of moderate heat stress on seed RFO accumulation. These experiments revealed a substantial 1.7‐fold variation in dry bean RFO levels due to both genetic and environmental factors and a significant increase in RFO content when plants encounter abiotic stress events during seed fill. Dry bean RFO content was positively correlated with sucrose levels but was uncorrelated with starch levels. When samples from the multi‐environment trial were subjected to canning, large varietal differences were discovered in the extent of RFO reduction in canned beans due to leaching, varying from 30% to 69% reductions from starting levels.

**CONCLUSION:**

This study clarifies the impact of environmental factors on RFO levels and identifies particular genotypes and market classes with low RFO levels. However, to predict dry bean crops that will contain lower RFO content in finished food products, variation in RFO leaching must also be considered. © 2025 His Majesty the King in Right of Canada. *Journal of the Science of Food and Agriculture* published by John Wiley & Sons Ltd on behalf of Society of Chemical Industry. Reproduced with the permission of the Minister of Agriculture and Agri‐Food.

## INTRODUCTION

Dry beans (*Phaseolus vulgaris*; common bean) are the world's most widely cultivated and consumed pulse crop, representing an economically accessible and environmentally sustainable source of protein across the globe.^1^, ^2^ They are generally considered to be an excellent source of nutrients including protein, complex carbohydrates, fiber and minerals.^1^, ^3^ However, they also contain high levels of raffinose family oligosaccharide (RFO), which is considered an antinutrient because it can cause flatulence and digestive discomfort.^4^ Reduction in RFO levels in raw dry beans and dry bean food products is therefore desirable to promote increased acceptability and consumption of dry beans within the Western diet.^5^

Raffinose family oligosaccharides are a group of small soluble oligosaccharides in which galactose molecules are attached in a chain through *α*(1 → 6) glycosidic linkages to a sucrose group.^6^ The typical RFO molecules are raffinose, stachyose, and verbascose, which contain one, two, and three galactose residues, respectively. Humans lack the *α*‐galactosidase enzyme necessary to cleave the *α*(1 → 6) glycosidic bond. Following ingestion, RFO is therefore not hydrolyzed and absorbed by the small intestine, but rather is rapidly metabolized by gut flora in the large intestine, resulting in gaseous hydrogen, methane and CO_2_ production, along with short‐chain fatty acids.^4^ Besides flatulence, the rapid production of these metabolic products can trigger intestinal discomfort and diarrhea, particularly in people with irritable bowel syndrome.^7^ For this reason, RFO is categorized as a fermentable oligosaccharide, disaccharide, monosaccharide and polyol (FODMAP) and is avoided by individuals subscribing to a low FODMAP diet.^8^

Raffinose family oligosaccharides are highly abundant in the seeds of many crops, particularly legumes, where they are synthesized from sucrose and galactinol by the enzymes raffinose synthase and stachyose synthase during the desiccation phase of seed development.^6^ The accumulation of RFOs likely contributes to the cellular and molecular stability of mature legume seeds and therefore plays a beneficial role in seed storage and longevity.^9^ Upon imbibition, RFOs also represent an immediately available carbohydrate supply that can be metabolized to fuel seed germination.^10^ They therefore provide benefits to the seed during development, storage, and germination.

Among pulses, there is a large variation in both the accumulation of total RFO and the types of RFOs that accumulate.^11^ In dry beans, stachyose is the dominant RFO, typically representing approximately 88% of total RFO, with minor contributions from raffinose (approximately 10%) and trace amounts of verbascose.^12^, ^13^ Sucrose is the other dominant soluble sugar in mature dry beans, with sucrose and stachyose together constituting approximately 90% of total soluble sugars.^14^ Substantial variation in RFO levels between dry bean varieties has been observed previously, with total RFO content ranging from 2.4% to 4.6% dry weight in greenhouse‐grown samples^13^ and from 3.5% to 6.0% dry weight in field‐grown samples.^12^ Another study focused on field‐grown yellow beans identified a lower range of RFO levels, from 1.5% to 3.4% dry weight.^15^

Environmental effects on RFO content in field‐grown dry bean have not been studied widely. A location‐dependent difference in mean RFO content was not observed following an assessment of a diverse dry bean panel grown at two field locations in the USA.^12^ By contrast, substantial environmental effects on RFO content were observed in two dry bean cultivars grown at contrasting altitudes in Italy.^16^ In other pulses, field studies have shown growth location effects on RFO accumulation, as in chickpea and lentil.^17^, ^18^ Implementation of controlled heat stress during seed fill in various crops is also known to affect seed nutritional properties.^19^, ^20^ In particular, heat and drought stress during seed fill affected the timing and extent of RFO accumulation in lupin.^21^

Raffinose family oligosaccharides can be strongly depleted in whole pulses by certain processing steps before consumption. Levels of RFOs are rapidly depleted upon sprouting because of internal catabolism.^22^ More commonly, soaking of legume seeds is an effective means of reducing RFO levels as they are highly water‐soluble and leach into the soaking liquid or are hydrolyzed by enzymes.^23^ Methods such as blanching and boiling, which increase the rate or extent of seed hydration, promote the leaching of RFOs.^23^ Due to these processes, canned dry beans contain lower levels of RFOs than raw beans but whether the extent of this reduction varies between dry bean varieties has not been reported.

This study assessed variations in dry bean RFOs, sucrose, and starch contents across a diverse panel of dry bean lines grown in multiple locations in Alberta, Canada, across three field seasons. It also investigated the effects of heat stress during seed fill on RFO accumulation in mature seeds and evaluated varietal differences in RFO depletion during canning. The findings provide insight into how factors affecting RFO levels in raw and processed dry beans can guide the selection of cultivars suited for producing foods with reduced RFO content.

## MATERIALS AND METHODS

### Dry bean lines

Twenty‐five dry bean genotypes representing six market classes – pinto, great northern, yellow, red, black, and cranberry – were assessed for carbohydrate content. An additional 12 genotypes (37 in total) were evaluated for carbohydrate leaching during canning. The genotypes included registered cultivars grown commercially under irrigation in western Canada and experimental lines from the dry bean breeding program at Agriculture and Agri‐Food Canada (AAFC), Lethbridge.

### Field trials

Dry bean genotypes were grown in performance yield trials at four locations in Alberta from 2021 to 2023: Fairfield Research Farm (Lethbridge County), Lethbridge On‐station, the Vauxhall substation of AAFC, and a grower field in Bow Island. Plots were managed according to established agronomic practices for commercial dry bean production.^24^ The experimental design was a randomized complete block with four replications per location. Each wide‐row plot consisted of four rows with rows spaced 0.6 m apart and 5 m in length. The planting density was 25 seeds m^−2^.

### Heat stress treatment under controlled growth conditions

The experimental design consisted of 12 dry bean varieties subjected to three different treatments with three replicate plants per variety per treatment. The entire experiment was repeated once. Two seeds were sown in 4 L pots containing Promix BX soil supplemented with 5 g per pot of N‐Charge inoculant (Verdesian Life Sciences, Cary, NC, USA) and 13.2% iron chelate at 0.3 g L^−1^. Pots were later thinned to one plant per pot. Plants were grown in a glasshouse under common conditions, consisting of natural light supplemented with 16 h of full spectrum light‐emitting diode (LED) lighting, day time temperature between 18 °C and 22 °C, night time temperature between 16 °C and 20 °C, watered on alternate days, and fertilized with 1 g L^−1^ of 20–20–20 (%NPK) once per week. After 52 days, when many pods reached the mid pod‐fill R6 stage,^25^ the plants were divided into three equal groups: control treatment, heat treatment 1, and heat treatment 2. Control and heat‐treatment 2 plants remained in the glasshouse under the above conditions. Heat treatment 1 plants were transferred immediately to a walk‐in growth cabinet for 10 days of heat treatment. Heat treatment conditions were 16 h of 150–200 μmol m^−2^ s^−1^ artificial light at the top of the canopy, with a day time temperature of 28 °C, night time temperature of 16 °C, and 300 mL of water per pot per day. After 10 days, heat treatment 1 plants were returned to the control conditions in the glasshouse and watered well. At this point, heat treatment 2 plants were transferred from the glasshouse to the growth cabinet for the same 10‐day heat treatment, then returned to the glasshouse and watered well. Afterwards, plant watering was stopped, and plants were left to dry. Dried beans from each plant were harvested manually in paper bags and dried in an oven at 40 °C for 34 h.

### Canning

Dry bean seed samples were collected for 37 genotypes from the four field trial locations in 2022 and 2023. Samples from each of the four replications per location were pooled to generate one composite canning sample per genotype per location. The moisture content of the seeds was determined using a GAC2500‐INTL Moisture Tester (Dickey‐John, Auburn, IL, USA), and 90 g of seed sample on a moisture‐free basis was subjected to canning.^26^ Hydration coefficient measurements were taken after 14 h soaking at 21 °C in deionized water. After a storage period of approximately 3 months, cans were opened and canned beans were placed in a colander and rinsed with water, then dried with paper towels. Beans were mashed in a mortar and pestle and 50 g of mashed beans were dried at 105 °C for 19 h. Samples were then ground to a fine powder and subjected to carbohydrate analysis.

### Carbohydrate and protein measurements

For carbohydrate measurements, 10 g samples of dry seed were ground to a fine powder, and 100 mg of powder was extracted twice in 1 mL of 80% (v/v) methanol for 15 min at 65 °C followed by centrifugation. For aqueous separation, 750 μL of combined supernatant was mixed with 750 μL water and 400 μL chloroform followed by centrifugation. Samples of the aqueous layer were subsequently dried in a vacuum centrifuge. Samples were resuspended in sodium acetate and the total RFO and sucrose content were measured using the raffinose/sucrose/d‐glucose assay kit (Megazyme, Bray, Ireland) following the manufacturer's instructions. Liberated glucose amounts attributable to RFO were converted to total RFO weights on a 100% stachyose basis. Starch measurements were conducted using the pellets obtained from each powder following the initial methanol extraction and centrifugation. The pellet was resuspended in 1500 μL of 40 mM sodium acetate buffer (pH 4.5) and starch content was measured using the total starch assay kit (Megazyme) following the manufacturer's protocol. Briefly, samples were digested with 30 U of amylase at 95 °C for 15 min while shaking. Next, 80 μL of the sample was digested with 20 U of amyloglucosidase for 40 min at 50 °C. Glucose was then quantified using the glucose oxidase‐coupled reaction.

Protein was measured directly from 30 mg of dry powdered sample following the Dumas method and using a Thermo Scientific FlashEA 1112 elemental analyzer. The conversion factor was 6.5. Three technical replicate powders were processed for each sample and mean values were calculated.

### High‐performance liquid chromatography

Dried samples were resuspended in 66% (v/v) acetonitrile in water with 50 mg mL^−1^ maltose as an internal standard. Samples were injected onto a Waters bridged ethylene hybrid (BEH) amide column (130 Å, 1.7 μm, 2.1 mm × 100 mm) maintained at 70 °C and connected to a Waters Acquity ultra‐performance liquid chromatography (UPLC) system equipped with photodiode array and evaporative light scattering (ELS) detectors. Carbohydrates were eluted over a 5 min linear gradient from 91.8% to 86.5% acetonitrile:water followed by a 15 min convex gradient from 86.5% to 62.5% acetonitrile:water. Chromatogram ELS peaks were integrated using Empower software (Waters, Milford, Massachustetts, USA), normalized to the internal standard, and quantified by interpolation against linear standard curves for sucrose, stachyose, and raffinose.

### Statistical analysis

For field trials, an analysis of variance (ANOVA) was conducted to compare the main effects of variety and environment (site‐year) for all variables using the Proc Mixed procedure in SAS Studio (SAS Institute, Cary North Carolina, USA). Replications nested within environment were considered as a random factor, and environment and variety as fixed effects. Significance among varieties and environments was determined using the least significant difference (LSD) mean comparison method (*P* ≤ 0.05). Broadsense heritability (H^2^) was calculated using the mean sum of squares from the ANOVA.^27^ For canned samples, ANOVA was conducted similarly with variety as a fixed effect and environment (growth location) as a random effect. For the heat treatment experiment, ANOVA included variety and treatment as fixed effects and experimental replicate as a random effect.

## RESULTS

### Enzymatic versus chromatographic analysis of total dry bean RFO

Soluble carbohydrates were quantified in 78 dry bean samples representing 26 lines from one site‐year using hydrophilic interaction chromatography (HILIC) and they were compared with the enzymatic determinations of sucrose and total RFO to evaluate the additional information provided by HILIC. As expected, the HILIC data showed that stachyose was the dominant RFO in all dry bean samples (Supporting Information, Fig. S1). Raffinose was a minor peak, and verbascose was below the reliable detection limit. Sucrose was the other major soluble sugar and free glucose levels were negligible. The primary trait of interest was total RFO, and individual RFO species provided limited additional information. When the quantities of raffinose and stachyose measured by HILIC were added together there was a moderate correlation with the enzymatic RFO measurements (Supporting Information, Fig. S1). However, averaging the three replicate samples for each variety produced a strong correlation between the two total RFO measurement methods (*R* ^2^ = 0.83). Sucrose measurements were also highly correlated between methods (Supporting Information, Fig. S1). Considering the lower cost and faster protocol, enzymatic determination of total RFO was appropriate for the large number of samples in this study.

### Genetic and environmental variability in dry bean RFO levels

The levels of total RFO, sucrose and starch were measured in 25 dry bean varieties grown at four locations in Alberta, with four randomized complete blocks per location, for three consecutive field seasons. There was significant variation in the content of all three carbohydrates due to genetic and environmental effects and their interaction (Table 1). When examined across all samples, there were no meaningful correlations among RFO, carbohydrate or sucrose levels (*R* ^2^ < 0.01). However, positive intra‐genotype correlations between RFO and sucrose amounts were evident in several lines, with an mean correlation of *R* = 0.38 (Supporting Information, Table S1). Seed weight and RFO content were modestly and consistently negatively correlated within varieties (*R* = −0.30; Supporting Information, Table S1).

**Table 1:** Summary statistics from analysis of variance of carbohydrate levels in a diverse panel of 25 dry bean genotypes across 12 site‐years

		RFO	Sucrose	Starch
Effect	DF	*p* value		
Variety	24	<0.0001	<0.0001	<0.0001
Environment	11	<0.0001	<0.0001	<0.0001
G × E	264	0.032	<0.0001	0.0007
Mean (g 100 g^−1^)		3.5	3.0	36.7
Range (g 100 g^−1^)		2.5–4.8	1.7–4.2	22.3–44.3
CV		10.6%	16.0%	7.5%
Broad sense heritability (H^2^)		0.94	0.98	0.90

Abbreviation: RFO, raffinose family oligosaccharide.

Among varieties/cultivars, there was a 39% difference between the highest (‘CDC Blackstrap’) and lowest (‘AAC Y073’) mean RFO levels, and the mean RFO content for all other lines was within 10% of the overall mean (Table 2). Mean sucrose levels varied by 58% between the highest and lowest lines, demonstrating proportionally more variation than RFO levels (Table 3). The mean starch content across lines varied less on a proportional basis but to a much greater extent on an absolute basis compared with RFO (Table 4). Relative differences in RFO, sucrose and starch content between varieties/cultivars demonstrated good repeatability across site‐years with heritability values (H^2^) above 0.9 (Table 1).

**Table 2:** Raffinose family oligosaccharide (RFO) levels in the dry bean diversity panel across 12 site‐years.

Variety/cultivar	RFO (g 100 g^−1^)													
	2022	2021	2021	2022	2022	2022	2021	2021	2023	2023	2023	2023	Avg	LSD_0.05_
	BI	BI	LE	LE	FF	VH	FF	VH	LE	FF	VH	BI		
‘AAC Y073’	2.73	2.93	3.04	2.91	2.50	2.51	3.01	3.23	3.42	2.96	3.30	3.78	3.03	0.18
‘AC Redbond’	3.26	3.10	2.77	3.01	3.11	3.15	3.28	3.05	3.08	3.24	3.63	3.67	3.20	0.14
‘AAC Y012’	3.13	2.91	2.94	3.05	3.13	2.94	3.15	3.47	3.55	3.54	3.33	3.93	3.26	0.21
‘AAC Cranford’	3.20	2.80	3.08	3.27	3.08	2.91	3.34	3.39	3.11	3.35	3.49	4.14	3.26	0.10
L18PS644	3.06	3.32	2.87	3.45	3.09	3.72	2.85	3.45	3.44	3.44	3.68	3.88	3.36	0.20
‘AAC Y015’	2.73	3.07	3.09	3.37	3.36	3.30	3.32	3.44	3.28	3.58	3.82	3.90	3.36	0.18
L19PS653	3.09	3.22	3.21	2.98	3.09	3.62	3.56	3.41	3.62	4.08	3.32	3.93	3.43	0.14
‘AAC PT601’	3.00	3.32	3.41	3.00	3.29	3.48	3.52	3.44	3.62	3.94	3.87	3.67	3.46	0.18
‘AAC Expedition’	3.23	3.14	3.26	3.33	3.40	3.53	3.31	3.64	3.59	3.61	3.67	4.27	3.50	0.21
‘AAC Whitehorse’	3.16	3.17	3.34	3.38	3.42	3.14	3.42	3.57	3.55	3.70	4.04	4.39	3.52	0.14
‘CDC Sunburst’	3.32	3.30	3.50	3.17	3.39	3.16	3.33	3.32	3.85	3.59	4.06	4.40	3.53	0.19
‘AAC PT600’	3.59	3.14	3.39	3.40	3.34	3.95	3.34	3.41	3.56	3.62	4.05	3.78	3.55	0.17
‘AAC Whitestar’	3.38	3.29	3.57	3.40	3.64	3.44	3.51	3.44	3.74	3.56	3.61	4.07	3.55	0.16
‘AAC Alberta North’	3.61	3.33	3.35	3.43	3.40	3.40	3.36	3.46	3.61	3.72	4.04	4.10	3.57	0.17
‘AAC Black Diamond 2’	3.42	3.56	3.24	3.30	3.37	3.67	3.45	3.53	3.93	3.73	3.32	4.39	3.57	0.20
L19YL203	3.11	3.43	3.42	3.56	3.35	3.49	3.70	3.77	3.62	3.69	3.79	4.25	3.60	0.19
‘Resolute’	3.40	3.39	3.53	3.17	3.40	3.76	3.73	3.60	3.28	3.78	3.84	4.59	3.62	0.21
L17GN964	3.16	3.31	3.44	3.33	3.54	3.69	3.84	3.64	3.57	3.60	4.07	4.29	3.62	0.12
‘Island’	3.46	3.22	3.46	3.65	3.67	3.66	3.55	3.67	3.43	4.11	3.56	4.19	3.64	0.16
‘AAC GN963’	3.53	3.31	3.30	3.34	3.58	3.45	3.66	3.66	3.87	3.72	4.15	4.07	3.64	0.16
‘CDC WM‐3’	3.34	3.33	3.53	3.74	3.35	3.29	3.50	3.62	4.42	4.04	3.76	4.24	3.68	0.16
‘AAC Explorer’	3.28	3.58	3.41	3.52	3.61	3.74	3.71	3.75	3.59	3.71	4.20	4.30	3.70	0.21
‘AC Black Diamond’	3.44	3.57	3.50	3.41	3.69	3.79	3.67	3.73	3.81	3.93	3.40	4.49	3.70	0.14
L19PS654	3.72	3.50	3.54	3.14	3.56	3.67	3.87	3.68	3.73	3.99	3.97	4.13	3.71	0.22
‘CDC Blackstrap’	3.76	4.06	3.62	4.19	4.16	4.26	4.08	4.12	4.52	4.27	4.55	4.83	4.20	0.13
**Mean**	3.28	3.29	3.31	3.34	3.38	3.47	3.48	3.54	3.63	3.70	3.78	4.15	3.53	
**LSD**_**0.05**_	0.14	0.13	0.16	0.21	0.15	0.15	0.21	0.09	0.18	0.18	0.17	0.16		

*Note*: Genotypes and environments are sorted from lowest to highest mean RFO content. RFO values are shaded as a heat map to illustrate patterns in variation. LSD_0.05_ is calculated for both within genotype and within environment variation. Abbreviations: LSD, least significant difference test. RFO, raffinose family oligosaccharide.

**Table 3:** Sucrose levels in the dry bean diversity panel across 12 site‐years.

Variety/cultivar	Sucrose (g 100 g^−1^)													
	2021	2021	2021	2021	2022	2022	2023	2022	2023	2023	2022	2023	Avg	LSD_0.05_
	BI	VH	FF	LE	BI	FF	LE	LE	BI	FF	VH	VH		
‘Island’	1.74	1.98	2.30	2.09	2.21	1.96	2.34	2.44	2.23	2.59	2.49	2.54	2.24	0.09
‘CDC Blackstrap’	1.99	1.91	2.13	1.96	2.15	2.58	2.52	2.30	2.18	2.15	2.66	2.69	2.27	0.10
L19PS653	2.08	2.04	2.14	2.24	2.25	2.34	2.39	2.35	2.77	2.60	2.50	2.72	2.37	0.10
L17GN964	2.28	2.27	2.42	2.72	2.44	2.56	2.42	2.45	2.77	2.66	2.63	2.95	2.55	0.13
‘AAC GN963’	2.39	2.38	2.71	2.63	2.32	2.36	2.52	2.56	2.68	2.67	2.73	2.97	2.58	0.12
‘AAC Whitehorse’	2.24	2.49	2.42	2.66	2.46	2.43	2.57	2.54	2.71	2.75	2.86	2.91	2.59	0.12
‘AAC Alberta North’	2.26	2.49	2.51	2.65	2.65	2.56	2.47	2.74	2.88	2.73	2.66	2.97	2.63	0.16
‘CDC Sunburst’	2.21	2.31	3.00	2.82	2.67	2.81	2.84	2.62	2.70	2.90	2.89	3.05	2.74	0.13
‘AAC Whitestar’	2.28	2.48	2.83	2.71	2.77	2.59	2.73	3.11	2.97	2.75	3.10	2.97	2.78	0.10
‘AC Redbond’	2.67	2.52	2.55	2.43	2.79	2.83	2.73	3.09	3.26	2.97	2.94	2.92	2.81	0.10
‘AAC Cranford’	2.69	2.54	2.88	2.74	2.35	2.96	2.69	3.10	2.85	2.73	3.06	3.27	2.82	0.17
‘AAC Explorer’	2.54	2.58	2.66	2.67	2.89	2.91	3.02	2.72	3.33	2.86	3.01	3.10	2.86	0.14
‘Resolute’	2.81	2.59	2.83	3.13	3.02	2.95	2.95	3.33	2.78	3.08	3.16	3.24	2.99	0.16
‘AC Black Diamond’	2.51	2.51	2.87	2.68	2.72	3.26	2.91	3.16	2.88	3.09	3.73	3.83	3.01	0.12
‘AAC Black Diamond 2’	2.63	2.85	2.61	2.84	2.85	3.21	3.25	3.12	3.24	3.23	3.73	3.59	3.10	0.13
‘CDC WM‐3’	2.55	2.82	3.09	3.25	3.20	2.97	3.07	3.02	3.19	3.44	3.39	3.55	3.13	0.17
‘AAC Expedition’	2.67	2.87	2.97	3.06	3.40	3.02	3.18	2.99	3.71	3.30	3.54	3.83	3.21	0.16
‘AAC Y012’	2.77	3.30	3.44	3.07	3.16	3.38	3.29	2.79	3.17	3.50	3.35	3.92	3.26	0.13
‘AAC PT600’	2.85	2.81	3.14	3.45	3.36	3.24	3.33	3.42	3.42	3.72	3.55	3.81	3.34	0.15
L19PS654	2.88	3.12	3.11	3.15	3.40	3.29	3.35	3.32	3.55	3.77	3.75	3.68	3.36	0.12
‘AAC Y073’	2.92	3.25	3.24	3.12	3.41	3.40	3.32	3.57	3.47	3.52	3.64	3.62	3.37	0.11
‘AAC Y015’	3.20	3.41	3.49	3.12	3.50	3.41	3.42	3.28	3.29	3.48	3.49	3.57	3.39	0.17
‘AAC PT601’	2.94	3.05	3.04	3.55	3.25	3.19	3.34	3.39	3.65	3.90	4.17	4.18	3.47	0.13
L18PS644	2.90	3.05	3.17	3.29	3.72	3.40	3.66	3.59	3.73	3.70	3.87	3.95	3.50	0.11
L19YL203	3.31	3.47	3.88	3.27	3.63	3.57	3.60	3.16	3.46	3.61	3.74	3.91	3.55	0.19
**Mean**	2.54	2.65	2.81	2.83	2.87	2.90	2.93	2.96	3.06	3.09	3.20	3.33	2.93	
**LSD**_**0.05**_	0.16	0.11	0.15	0.16	0.15	0.10	0.15	0.15	0.13	0.10	0.13	0.12		

*Note*: Genotypes and environments are sorted from lowest to highest average sucrose content. Sucrose values are shaded as a heat map to illustrate patterns in variation. LSD_0.05_ is calculated for both within genotype and within environment variation. Abbreviation: LSD, least significant difference test.

**Table 4:** Starch levels in the dry bean diversity panel across 12 site‐years.

Variety/cultivar	Starch (g 100 g^−1^)													
	2022	2022	2021	2023	2023	2021	2022	2023	2021	2023	2022	2021	Avg	LSD_0.05_
	BI	VH	VH	LE	VH	FF	LE	FF	BI	BI	FF	LE		
‘AC Black Diamond’	22.3	31.8	34.7	35.1	35.1	36.2	33.5	35.5	36.1	34.9	34.1	34.3	33.6	1.3
‘CDC Blackstrap’	25.8	33.0	32.3	34.5	35.6	34.1	34.3	35.5	33.7	34.6	36.5	36.0	33.8	1.3
‘CDC Sunburst’	32.0	35.6	35.8	32.5	32.5	35.7	32.0	32.7	36.3	38.5	34.8	40.3	34.9	1.7
‘AAC Cranford’	35.3	36.1	33.9	31.6	32.8	36.8	37.5	31.8	37.9	37.2	37.6	37.8	35.5	1.3
‘CDC WM‐3’	34.1	30.1	36.5	34.1	36.0	34.7	36.8	37.0	35.2	38.1	37.4	36.6	35.6	1.5
‘AAC Y073’	33.4	33.1	35.4	35.7	33.4	36.9	37.2	37.1	36.5	36.7	36.2	36.2	35.7	1.4
‘AAC Y012’	32.6	29.6	35.4	35.4	37.2	34.5	36.9	37.2	36.8	37.6	38.2	37.8	35.8	1.7
‘AC Redbond’	35.1	36.5	35.2	36.0	33.5	36.5	35.0	36.4	36.9	37.0	35.4	36.2	35.8	1.2
‘Resolute’	30.1	38.7	36.1	31.9	35.3	36.3	35.1	37.8	38.7	37.5	35.7	37.9	35.9	1.4
L19YL203	33.5	36.0	34.9	33.9	35.8	37.0	37.3	35.3	35.7	36.9	36.3	38.9	36.0	1.5
‘AAC Y015’	34.6	30.7	34.6	36.0	35.7	36.6	36.6	38.6	36.2	36.6	37.9	37.5	36.0	1.9
‘AAC Whitestar’	30.2	33.7	35.9	36.2	37.4	37.7	35.5	35.9	36.7	36.7	36.8	39.6	36.0	1.6
‘AAC Black Diamond 2’	34.9	34.9	34.2	37.1	37.0	32.9	35.1	36.7	37.2	36.8	37.9	38.1	36.1	1.3
‘AAC Explorer’	37.5	35.0	36.2	30.0	37.6	36.1	36.7	36.6	36.9	37.0	38.1	39.2	36.4	1.2
‘AAC Alberta North’	33.0	32.3	36.7	37.2	38.5	37.4	37.1	36.3	39.0	38.6	38.7	36.4	36.8	1.5
‘AAC Expedition’	35.9	34.6	35.9	38.0	40.2	37.3	37.7	37.7	37.2	37.2	38.9	37.9	37.4	1.5
L17GN964	30.2	36.5	35.9	36.5	38.0	38.5	40.0	38.3	38.7	38.8	38.9	39.7	37.5	1.5
‘AAC Whitehorse’	30.1	38.8	35.9	38.2	37.9	39.4	36.8	36.5	38.4	37.5	39.2	41.8	37.5	1.7
L19PS654	33.4	36.3	36.7	39.2	35.9	36.3	38.0	38.7	38.4	39.1	40.5	40.6	37.8	0.9
‘AAC GN963’	39.7	36.1	36.1	36.0	38.7	37.2	36.9	39.0	38.6	39.4	37.4	42.4	38.1	1.5
L19PS653	35.2	39.8	38.2	40.5	39.3	38.4	38.3	39.8	37.7	40.1	40.8	40.1	39.0	1.5
‘AAC PT601’	29.6	38.8	37.4	44.4	38.8	38.0	38.6	39.9	40.4	39.5	40.8	41.9	39.0	1.4
‘AAC PT600’	32.5	36.2	37.7	39.7	38.6	37.9	38.9	41.5	39.8	41.0	41.9	42.9	39.1	1.4
L18PS644	38.1	34.4	37.1	38.7	40.4	39.8	40.3	41.0	39.2	39.4	40.9	40.9	39.2	1.4
‘Island’	38.0	39.4	37.9	40.2	39.1	39.9	40.1	40.3	39.1	40.1	42.0	39.3	39.6	1.1
**Mean**	33.1	35.1	35.9	36.4	36.8	36.9	36.9	37.3	37.5	37.9	38.1	38.8	36.7	
**LSD**_**0.05**_	2.2	1.9	0.8	1.2	1.3	1.3	1.5	1.1	0.8	0.8	1.1	2.0		

*Note*: Genotypes and environments are sorted from lowest to highest mean starch content. starch values are shaded as a heat map to illustrate patterns in variation. LSD_0.05_ is calculated for both within genotype and within environment variation. Abbreviation: LSD, least significant difference test.

Environmental variation in RFO content was particularly evident in one site‐year, Bow Island (BI) 2023, which suffered a hail storm on 10 August 2023, resulting in damage to the canopy and pods, which were at R7 and R8 growth stages (Table 2). This stress event coincided with a significant rise in RFO, but not starch or sucrose, in comparison with all other site‐years.

To compare RFO, sucrose and starch variation amongst market classes, the data were reorganized (Supporting Information, Tables S2–4). Yellow beans tended to have lower RFO levels, while the single entries for market classes Red and Cranberry also had low mean RFO levels (Supporting Information, Table S2). Yellow and Pinto beans tended to have higher mean sucrose levels (Supporting Information, Table S3). Pinto beans tended to have higher starch content and black beans had lower starch content (Supporting Information, Table S4).

### Heat stress during seed fill induces RFO accumulation

**Figure 1:**
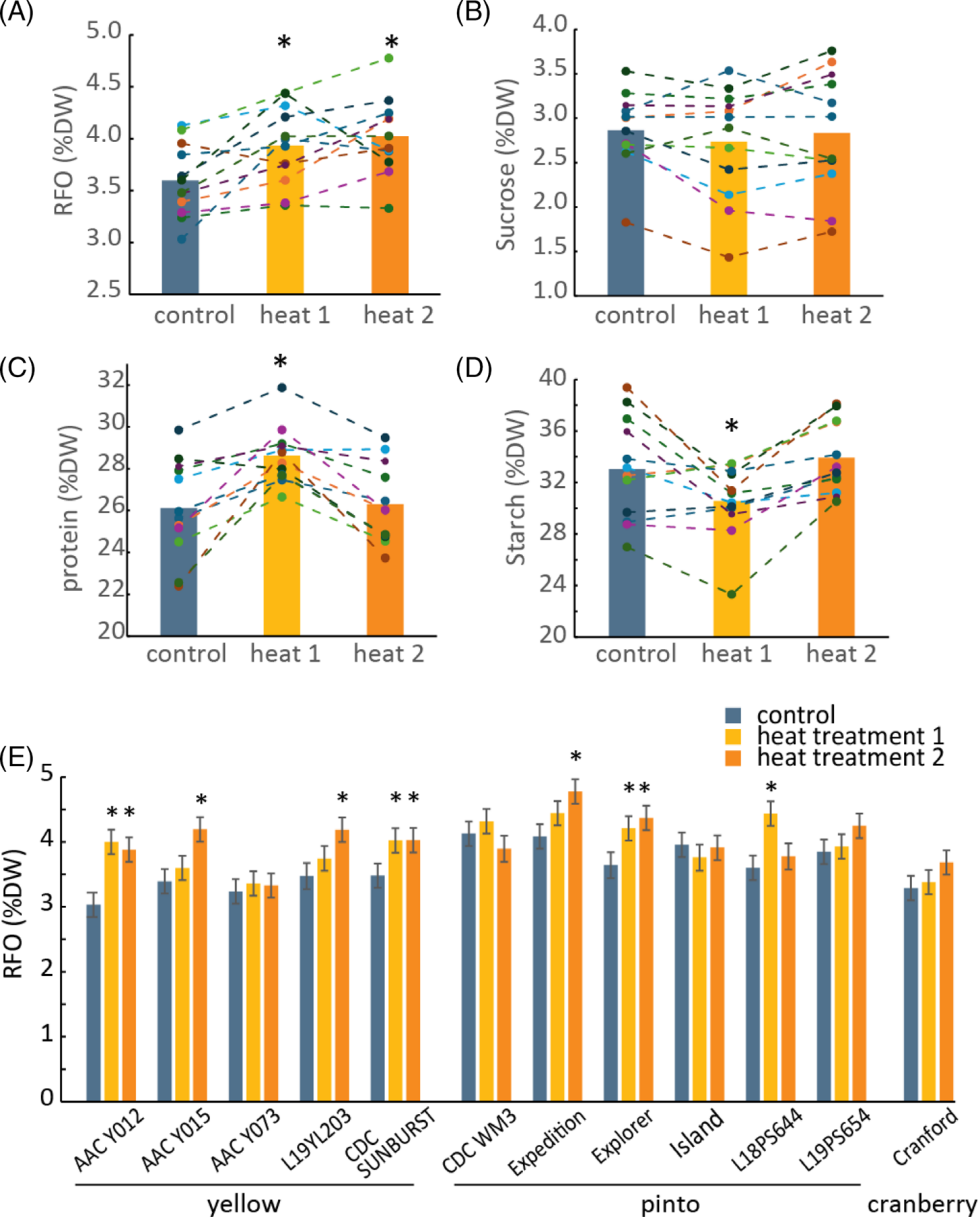
Effect of heat treatment during seed fill on dry bean nutrient composition. (A–D) Bars indicate the overall mean concentrations of (A) raffinose family oligosaccharide (RFO), (B) sucrose, (C) protein and (D) starch across all varieties following control or heat treatments. Data points are variety means, which are connected between treatments to illustrate trends. Asterisks indicate significant differences between means of treatment and control groups (*P* < 0.05). (E) Raffinose family oligosaccharide (RFO) concentrations for each variety are indicated and sorted by market class. Asterisks indicate significant differences between treatment and control means within a genotype (*P* > 0.05; LSD = 0.45; *n* = 6). Error bars indicate standard error of the mean. LSD, least significant difference test.

We next investigated the effect of heat stress during seed fill on the accumulation of RFO. Replicate greenhouse‐grown plants from 12 dry bean varieties were subjected to one of two sequential 10 day heat treatments during seed fill (28 °C daytime temperature) or left under control conditions (22 °C daytime temperature). Seeds were characterized at maturity for RFO, sucrose, starch and protein content. Across all lines, heat treatments 1 and 2 increased mean seed RFO content by 9 and 12%, respectively in comparison with the control treatment (Fig. 1(A)). There was also a significant variety‐by‐treatment interaction as seven of the 12 varieties displayed significant RFO increases upon heat treatment, with the remaining five varieties showing no change (Fig. 1(E)). The most sensitive cultivar was ‘AAC Y012’, which accumulated 30% more RFO on average in response to heat (Fig. 1(E)). Mean protein content across all varieties increased by 10% in heat treatment 1 but was unaffected by heat treatment 2 (Fig. 1(C)). The opposite effect was observed with starch, which decreased by 8% on average in heat treatment 1 (Fig. 1(D)), and there was a negative intra‐genotype correlation between protein and starch levels, with an mean correlation of *R* = −0.5 (Supporting Information, Table S5). Sucrose levels were only marginally affected by heat treatments and no consistent pattern was observed across genotypes (Fig. 1(B)). The individual measurements of sucrose, starch and protein levels for individual varieties following heat treatments are shown in Supporting Information, Fig. S2. Overall, the increase in RFO content was the most consistent response to heat treatment during seed fill among the nutrients measured.

### Nutrient leaching is variable across dry bean genotypes

During the canning process, the RFO content within dry beans decreases due to leaching of RFO into surrounding liquid. However, variation in RFO reduction among canned dry bean varieties has not been examined. This study investigated the effect of canning on 37 dry bean varieties from the four different field trial growth locations in 2022 and 2023. There were significant differences in the mean RFO abundances among varieties in both unprocessed dry seeds and canned samples (Table 5). There was notably no correlation between the raw and canned RFO measurements (Table 6). In other words, there was marked variation in the proportion of RFO leaching upon canning between varieties, ranging from 43% to 69% of total RFO (Table 5). Canned RFO content was largely determined by RFO leaching and not by starting RFO content in raw beans (Table 6). Unlike RFO levels, sucrose levels correlated well between dry seed and canned samples (Table 6). Although sucrose and RFO leaching were positively correlated there were differences between them in the extent of leaching, indicating that leaching processes can differ between soluble carbohydrates. Seed hydration coefficients were positively correlated with sucrose and RFO leaching (Table 6). Finally, sucrose leaching, and to a lesser extent RFO leaching, were negatively correlated with seed size, suggesting that the larger seeds retain proportionally more soluble carbohydrates upon canning.

**Table 5:** Raffinose family oligosaccharide (RFO) and sucrose quantities in canned dry bean compared to unprocessed samples.

Variety	Market Class	Mean RFO			Mean sucrose		
		Canned (%DW)	Dry seed (% DW)	Leaching (%)	Canned (%DW)	Dry seed (% DW)	Leaching (%)
‘AAC Y073’	Yellow	1.66	2.98	42.7	2.04	3.49	41.7
‘AAC Cranford’	Cranberry	1.80	3.29	44.6	2.01	2.89	30.4
‘CDC Sunburst’	Yellow	1.85	3.53	46.5	1.91	2.82	31.6
L17GN964	GN	1.92	3.65	46.9	1.32	2.60	48.8
L20YL238	Yellow	1.65	3.22	48.1	2.29	3.65	36.9
L20YL236	Yellow	1.70	3.38	49.3	1.59	2.99	46.2
L18PS644	Pinto	1.72	3.42	49.4	1.98	3.75	46.7
‘AAC Whitehorse’	GN	1.76	3.61	50.3	1.25	2.67	52.7
L19PS653	Pinto	1.69	3.47	50.4	1.47	2.49	40.3
L20YL258	Yellow	1.60	3.26	50.4	1.94	3.78	48.1
L19YL203	Yellow	1.74	3.58	50.5	2.18	3.59	38.7
L20YL253	Yellow	1.83	3.78	50.9	1.55	2.74	43.2
L20YL256	Yellow	1.63	3.40	51.1	1.90	3.19	40.1
L20PS716	Pinto	1.73	3.58	51.2	1.91	3.33	41.8
‘AAC Y012’	Yellow	1.57	3.27	51.3	1.85	3.34	43.5
‘AAC PT600’	Pinto	1.76	3.68	52.0	2.03	3.48	41.3
‘AAC Y015’	Yellow	1.58	3.34	52.1	1.83	3.47	46.9
‘AC Black Diamond’	Shiny Black	1.77	3.74	52.4	1.70	3.20	46.2
‘AAC GN963’	GN	1.74	3.68	52.4	1.31	2.61	49.0
L20PS737	Pinto	1.51	3.22	52.6	1.49	2.73	44.5
‘AAC Alberta North’	GN	1.73	3.66	52.8	1.22	2.71	55.1
L19PS654	Pinto	1.72	3.68	52.9	1.85	3.56	47.7
L20GN992	GN	1.64	3.62	54.1	1.35	2.76	50.5
‘Resolute’	G.N.	1.64	3.66	54.3	1.34	3.06	56.0
‘AAC Black Diamond 2’	Shiny Black	1.65	3.66	54.4	1.74	3.28	46.6
‘AAC PT601’	Pinto	1.56	3.49	54.7	1.93	3.58	44.8
L20PS739	Pinto	1.64	3.64	54.9	1.33	2.68	50.1
‘AAC Whitestar’	GN	1.62	3.62	55.2	1.15	2.88	59.9
L20PS742	Pinto	1.57	3.76	56.6	1.45	3.32	55.5
‘Island’	Pinto	1.58	3.70	57.0	1.25	2.38	46.4
L20PS713	Pinto	1.55	3.70	57.7	1.61	3.35	51.4
‘AC Redbond’	Red	1.35	3.31	59.0	1.41	2.97	52.2
L20RE745	Red	1.40	3.50	59.4	1.36	2.96	53.9
‘AAC Expedition’	Pinto	1.38	3.57	61.3	1.35	3.37	59.3
‘CDC WM‐3’	Pinto	1.37	3.72	62.9	1.28	3.25	60.4
‘CDC Blackstrap’	Matte Black	1.43	4.31	66.6	1.00	2.40	58.1
‘AAC Explorer’	Pinto	1.14	3.71	69.0	0.93	3.01	68.8
**Mean**		1.63	3.55	53.5	1.60	3.09	48.0
**Range**		1.14–1.92	2.98–4.31	43–69	0.93–2.29	2.38–3.77	30–69
**CV**		9.8	6.4	10.3	21.6	12.8	16.8
**LSD**_**0.05**_		0.23	0.21	7.3	0.20	0.18	6.8

*Note*: Mean RFO and sucrose values are calculated as percent dry weight from samples from four locations each in 2022 and 2023. Leaching percentage was calculated from canned and dry seed values as the proportion of RFO or sucrose that was lost during the canning process. All parameters varied significantly among varieties *P* < 0.0001 and LSD_0.05_ is shown.

Abbreviations: GN, Great Northern. LSD, least significant difference test.

**Table 6:** Correlation coefficients across varieties for traits associated with dry bean canning

	RFO canned	Sucrose canned	RFO dry seed	Sucrose dry seed	RFO leaching	Sucrose leaching	Hydration coefficient
Sucrose canned	**0.43**						
RFO dry seed	−0.06	**−0.57**					
Sucrose dry seed	−0.06	**0.71**	**−0.40**				
RFO leaching	**−0.83**	**−0.67**	**0.59**	−0.16			
Sucrose leaching	**−0.66**	**−0.80**	**0.47**	−0.16	**0.80**		
Hydration coefficient	**−0.47**	**−0.74**	**0.44**	−0.28	**0.62**	**0.79**	
Seed weight	0.28	**0.41**	**−0.56**	0.16	**−0.54**	**−0.46**	−0.29

*Note*: bold values indicate significant correlations with *P* < 0.05. *n* = 37.

Abbreviation: RFO, raffinose family oligosaccharide.

## DISCUSSION

### Variation in RFO levels during field trials

Soluble carbohydrate concentrations were compared using hydrophilic interaction chromatography (HILIC) and an enzymatic method. The correlation between methods was lower for total RFOs (*R* ^2^ = 0.49) than for sucrose (*R* ^2^ = 0.76) (Supporting Information, Fig. S1). The reduced RFO correlation likely reflected propagation of technical error, as both total RFO measurements combined two components: stachyose and raffinose for HILIC, and sucrose and sucrose plus RFO for the enzymatic assay. Averaging the three replicate samples per variety within a site–year improved agreement between methods, yielding a strong correlation (*R* ^2^ = 0.83).

The multi‐environment evaluation of RFO contents among a Western Canadian dry bean diversity panel produced a range of RFO values demonstrating substantial genetic and environmental variation (Table 1). The range of mean RFO content by dry weight among variety means in this study (3.0–4.2%) was consistent with the range observed by Kleintop *et al*.^13^ (2.4–4.6%) but lower than the range reported by Brick *et al*.^12^ (3.5–6.0%). Heritability for total RFO content was high (*H^2^* = 0.94), exceeding previous estimates for other legumes (0.45–0.74).^17^, ^28^, ^29^

‘CDC Blackstrap’ exhibited the highest RFO content across all 12 site‐years, averaging 13% more than the next highest entry. Similarly, Brick *et al*.^12^ identified a black‐seeded genotype with the highest RFO levels, suggesting that black beans may generally possess higher RFO content.^30^ Yellow beans, by contrast, were among those with the lowest RFO levels, consistent with a previous report of reduced RFO content (1.5–3.4% dry weight) in a diverse yellow bean panel.^15^ These findings indicate that yellow beans may be well suited for applications requiring low RFO levels. Additional entries from the red and cranberry market classes should be evaluated to determine whether they also exhibit consistently low RFO content. Overall, RFO concentrations overlapped among market classes, as previously reported,^30^ indicating that individual varietal evaluation remains necessary to estimate RFO levels accurately.

This study also investigated environmental variation in mean RFO content with site‐year means ranging from 3.3% to 3.8% by dry weight, excluding the hail‐damaged site, which showed a higher mean of 4.2% (Table 2). In lentils, modest environmental variation in field‐grown RFO levels was inversely associated with precipitation.^18^ In the present study, dry beans were grown under irrigation, and no correlation was observed between RFO content and temperature at any point in the growing season. However, under controlled conditions simulating a late‐season heat event during seed fill, elevated temperature increased RFO accumulation (Fig. 1). A similar positive association between temperature and RFO content during maturity has been reported in lupins.^21^

The hailstorm at Bow Island in 2023 accelerated crop maturation and resulted in unusually high RFO levels. It is therefore proposed that rapid maturation of hail‐damaged plants, which produced smaller seeds, contributed to the increased proportional RFO content. Supporting this interpretation, modest negative intragenotype correlations were detected between seed size and RFO levels, but not sucrose or starch, in the field trials (Supporting Information, Table S1). Overall, abiotic stresses during seed fill, such as heat or physical damage, may enhance RFO accumulation through a shared mechanism involving accelerated seed maturation. Further investigation is warranted to clarify the link between abiotic stress and RFO synthesis, which may involve enhanced stress signaling (e.g. abscisic acid) similar to that observed in vegetative tissues.^31^

### Variation in RFO during canning trials

It is well‐understood that soaking and canning lead to a depletion of RFO levels in pulses because of leaching.^32^ However, one finding of this study was that different dry bean cultivars displayed markedly different carbohydrate leaching properties in response to canning (Table 5). These differences were apparent at the level of market class, with yellow beans showing consistently less leaching than pinto beans. Thus some cultivars like ‘CDC Blackstrap’ with high RFO content before canning ended up with below‐average RFO content after canning. On the other hand, yellow cultivar ‘AAC Y073’ had the lowest RFO before canning and above‐average RFO after canning. Differences in sucrose leaching between cultivars were also observed. Less sucrose leaching was observed on average compared to RFO leaching, but the two values were well‐correlated. The reason for the varietal differences in RFO and sucrose leaching can be partly explained by considering other seed parameters as both leaching measurements were positively correlated with hydration coefficient and negatively correlated with seed size. One might expect that, following multi‐day storage in cans, all bean material would become fully hydrated and therefore equilibrated with the surrounding liquid. However, seeds with low hydration coefficients (<1.8) may not become fully hydrated even following canning and therefore display less leaching. Seeds with hydration coefficients greater than 2.2 may experience loss of seed coat integrity, thus removing a potential barrier to leaching. Additional unresolved mechanisms may be involved that differentiate the leaching potential between cultivars. Nevertheless, protocols for canning could be verified on a variety‐by‐variety basis in terms of maximizing RFO leaching.

### Correlations between nutrient classes

Raffinose family oligosaccharides have previously been measured alongside soluble and insoluble fiber; however, no correlation between RFO and other fiber classes has been observed.^30^ This study noted an intra‐genotypic correlation between sucrose and RFO levels (Supporting Information, Table S1), which is likely related to sucrose being a substrate for RFO biosynthesis. Positive correlations between substrates for the RFO pathway and RFO amounts were previously observed in chickpea.^17^ Conversely, a relationship between RFO and starch was not observed, suggesting that the accumulation of both compounds is regulated independently. By comparison, the study found a clear negative intra‐genotypic correlation between starch and protein in samples from our heat treatment experiment, suggesting that carbon diverted away from starch upon heat stress becomes utilized for protein synthesis. These results indicate that breeding efforts to lower RFO may also lower sucrose content but are unlikely to strongly affect protein or starch levels.

## CONCLUSIONS

This study examined sources of variation in carbohydrate content in dry beans to clarify factors influencing the reduction of RFO content in whole beans. The experiments demonstrated substantial RFO variation due to genetic effects, environmental effects, processing effects, and their interactions. Although varietal differences in dry bean RFO levels and RFO reductions upon soaking and canning have been reported previously, this study identified additional major sources of variation including environmental variation due to abiotic stress and varietal variations in the extent of RFO leaching during canning. Both of these effects were partially related to seed size.

In raw dry beans, RFO content showed little differentiation among market classes, whereas canned beans displayed clear market class differences. Levels of RFO in raw and canned beans were not correlated. Therefore, depending on end use, the variety/cultivar of choice to achieve low RFO is different, with yellow beans tending to have lower RFO in raw samples and pinto beans having lower RFO in canned samples. Finally, bean crops that are able to mature and desiccate in the absence of major abiotic stress events are likely to contain fully developed seeds with lower RFO content.

## AUTHOR CONTRIBUTIONS

Parthiba Balasubramanian and Brendan M. O'Leary conceived the research and designed the experiments. Parthiba Balasubramanian and Todd Reid conducted the field trial and provided samples. Brendan M. O'Leary and Vinti Kumari performed the other experiments. All authors contributed to data analysis. Brendan M. O'Leary wrote the manuscript with help from all authors.

## CONFLICT OF INTEREST

The authors declare no conflict of interest.

## Supporting information


**Figure S1.** HILIC analysis of dry bean carbohydrates. (A) Representative chromatogram of dry bean sugars following HILIC. (B, C) Correlation of total RFO or sucrose measurements between enzymatic‐based and HILIC‐based quantification of 78 samples (3 replicates of 26 varieties) from one site year. HILIC measurements are the mean of two technical replicates (*i.e*. the entire extraction and quantification was performed twice). Enzymatic measurements are the mean of three technical replicates. (D, E) Mean RFO or sucrose values for 26 varieties measured in panel B, C.
**Figure. S2.** Effect of heat treatment on seed nutrient composition. (A–C) The mean concentration of sucrose (A), protein (B) and starch (C) for each variety are shown for control, heat‐treatment 1 and heat‐treatment 2 plants, sorted by market class. Asterisks indicate significant difference between treatment and control means within a genotype (*P* > 0.05; *n* = 6). Error bars indicate standard error of the mean.
**Table S1.** Intra‐genotype correlation coefficients for carbohydrate contents from a dry bean diversity panel across 12 site‐years. Bold lettering indicates a significant correlation (*P* < 0.05, *n* = 12).
**Table S2.** RFO levels shown as percent dry weight in a dry bean diversity panel across 12 site‐years organized by market class. The ranking of mean RFO content for each genotype is indicated in superscript.
**Table S3.** Sucrose levels shown as percent dry weight in a dry bean diversity panel across 12 site‐years organized by market class. The ranking of mean sucrose content for each genotype is indicated in superscript.
**Table S4.** Starch levels shown as percent dry weight in a dry bean diversity panel across 12 site‐years organized by market class. The ranking of mean starch content for each genotype is indicated in superscript.
**Table S5.** Intra‐genotype correlations among nutrient classes across all treatments during the heat stress experiment. Bold indicates a statistically significant correlation (*P* < 0.05, *n* = 18).

## Data Availability

The data that supports the findings of this study are available in the supplementary material of this article.
