# Supplementary material for: Multi‐environment evaluation of raffinose‐family oligosaccharide content in diverse dry bean varieties and their reduction upon canning
Source: J Sci Food Agric. 2025 Nov 12;106(3):1904–13. doi: 10.1002/jsfa.70303 (PMC12790645; doi:10.1002/jsfa.70303)
Supplement: Supplementary file 1 — Figure S1. HILIC analysis of dry bean carbohydrates. (A) Representative chromatogram of dry bean sugars following HILIC. (B, C) Correlation of total RFO or sucrose measurements between enzymatic‐based and HILIC‐based quantification of 78 samples (3 replicates of 26 varieties) from one site year. HILIC measurements are the mean of two technical replicates (i.e. the entire extraction and quantification was performed twice). Enzymatic measurements are the mean of three technical replicates. (D, E) Mean RFO or sucrose values for 26 varieties measured in panel B, C. Figure. S2. Effect of heat treatment on seed nutrient composition. (A–C) The mean concentration of sucrose (A), protein (B) and starch (C) for each variety are shown for control, heat‐treatment 1 and heat‐treatment 2 plants, sorted by market class. Asterisks indicate significant difference between treatment and control means within a genotype (P > 0.05; n = 6). Error bars indicate standard error of the mean. Table S1. Intra‐genotype correlation coefficients for carbohydrate contents from a dry bean diversity panel across 12 site‐years. Bold lettering indicates a significant correlation (P < 0.05, n = 12). Table S2. RFO levels shown as percent dry weight in a dry bean diversity panel across 12 site‐years organized by market class. The ranking of mean RFO content for each genotype is indicated in superscript. Table S3. Sucrose levels shown as percent dry weight in a dry bean diversity panel across 12 site‐years organized by market class. The ranking of mean sucrose content for each genotype is indicated in superscript. Table S4. Starch levels shown as percent dry weight in a dry bean diversity panel across 12 site‐years organized by market class. The ranking of mean starch content for each genotype is indicated in superscript. Table S5. Intra‐genotype correlations among nutrient classes across all treatments during the heat stress experiment. Bold indicates a statistically significant correlation (P < 0.05, n = [file JSFA-106-1904-s001.docx]

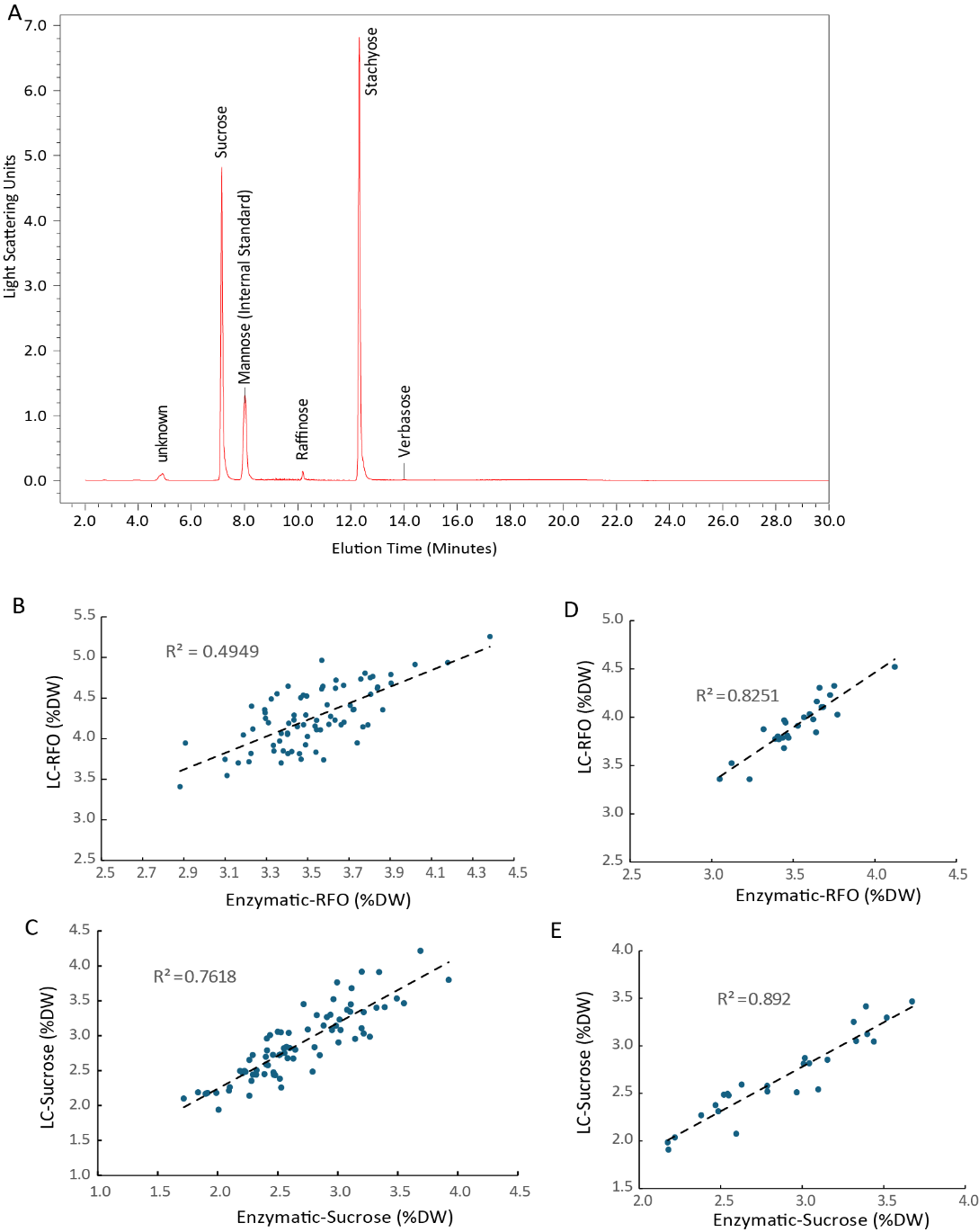


**Supplemental Figure 1: HILIC analysis of dry bean carbohydrates.**

A) Representative chromatogram of dry bean sugars following HILIC.

B-C) Correlation of total RFO or sucrose measurements between enzymatic-based and HILIC-based quantification of 78 samples (3 replicates of 26 varieties) from one site year. HILIC measurements are the mean of two technical replicates (*i.e.* the entire extraction and quantification was performed twice). Enzymatic measurements are the mean of three technical replicates.

D-E) Mean RFO or sucrose values for 26 varieties measured in panel B-C.


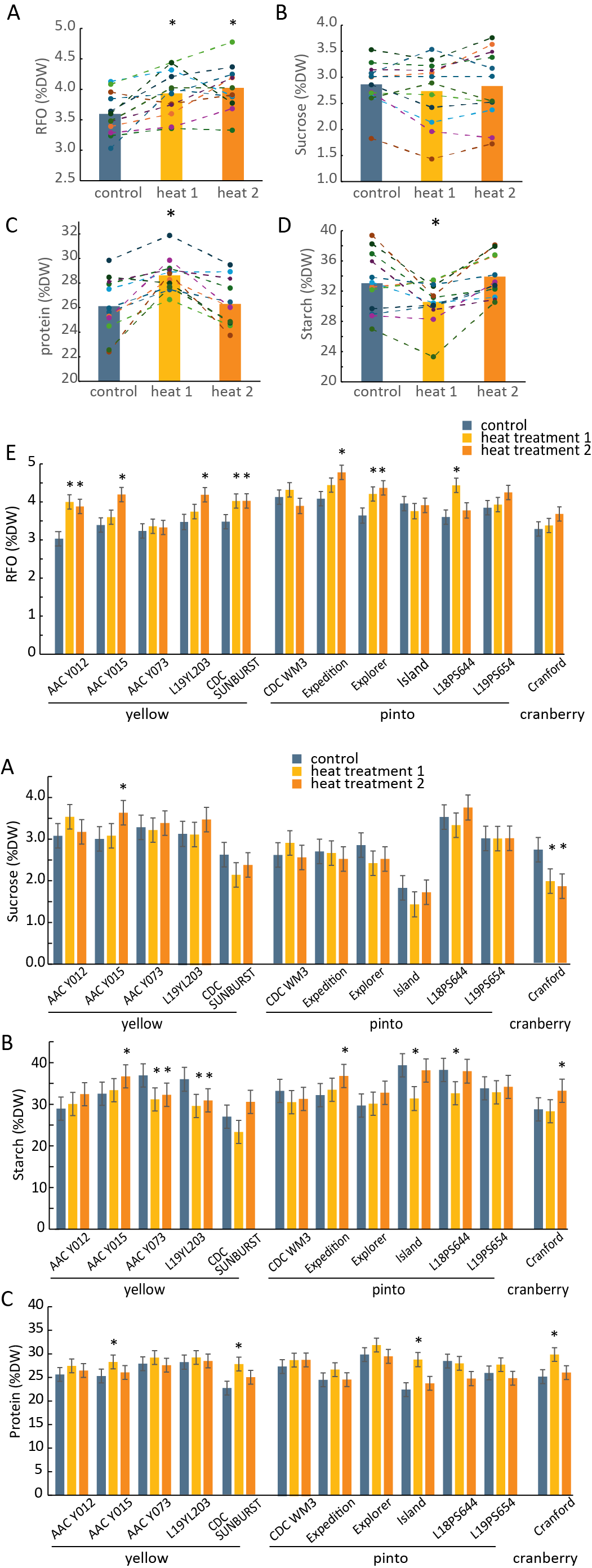


**Supplemental Figure 2:** Effect of heat treatment on seed nutrient composition. A-C) The mean concentration of sucrose (A), protein (B) and starch (C) for each variety are shown for control, heat-treatment 1 and heat-treatment 2 plants, sorted by market class. Asterisks indicate significant difference between treatment and control means within a genotype (p>0.05; n=6). Error bars indicate standard error of the mean.

**Supplemental Table 1**: Intra-genotype correlation coefficients for carbohydrate contents from a dry bean diversity panel across twelve site-years. Bold lettering indicates a significant correlation (*p*<0.05, n=12).

|  | Correlation Coefficient | | | |
| --- | --- | --- | --- | --- |
| **Genotype** | RFO:Sucrose | RFO:Starch | Sucrose:Starch | RFO:Seed wt |
| **Pinto** |  |  |  |  |
| ‘AAC Expedition’ | **0.64** | 0.06 | 0.12 | -0.40 |
| ‘AAC Explorer’ | **0.63** | 0.00 | -0.20 | 0.01 |
| ‘CDC WM-3’ | 0.23 | 0.34 | -0.16 | -0.09 |
| ‘Island’ | 0.43 | 0.33 | 0.12 | -0.09 |
| ‘AAC PT600’ | **0.78** | -0.19 | 0.08 | -0.54 |
| ‘AAC PT601’ | 0.55 | 0.46 | 0.10 | -0.15 |
| L18PS644 | 0.53 | -0.36 | -0.12 | -0.41 |
| L19PS653 | 0.52 | 0.40 | 0.45 | -0.15 |
| L19PS654 | 0.49 | -0.17 | -0.19 | -0.13 |
|  |  |  |  |  |
| **Great Northern** |  |  |  |  |
| ‘AAC Whitehorse’ | 0.48 | 0.07 | 0.15 | -0.44 |
| ‘AAC Whitestar’ | 0.29 | 0.33 | -0.17 | 0.14 |
| ‘AAC GN963’ | 0.51 | -0.11 | 0.11 | -0.24 |
| L17GN964 | **0.60** | 0.33 | 0.26 | -0.17 |
| ‘AAC Alberta North’ | **0.75** | 0.22 | -0.07 | **-0.58** |
| ‘Resolute’ | -0.26 | 0.40 | -0.08 | **-0.64** |
|  |  |  |  |  |
| **Yellow** |  |  |  |  |
| ‘AAC Y012’ | 0.33 | 0.29 | -0.06 | -0.19 |
| ‘AAC Y015’ | 0.20 | 0.18 | -0.33 | -0.06 |
| ‘AAC Y073’ | -0.07 | 0.23 | -0.38 | -0.15 |
| ‘CDC Sunburst’ | 0.30 | 0.14 | -0.18 | -0.55 |
| L19YL203 | 0.11 | 0.21 | -0.34 | -0.09 |
|  |  |  |  |  |
| **Black** |  |  |  |  |
| ‘AC Black Diamond’ | -0.09 | 0.28 | 0.04 | **-0.62** |
| ‘AAC Black Diamond 2’ | 0.24 | 0.10 | 0.24 | -0.54 |
| ‘CDC Blackstrap’ | 0.48 | 0.36 | 0.23 | -0.21 |
|  |  |  |  |  |
| **Cranberry** |  |  |  |  |
| ‘AAC Cranford’ | 0.11 | -0.12 | 0.11 | -0.55 |
|  |  |  |  |  |
| **Red** |  |  |  |  |
| ‘AC Redbond’ | **0.61** | -0.15 | -0.02 | -0.37 |
|  |  |  |  |  |
| **Mean** | 0.38 | 0.15 | -0.01 | -0.29 |

**Supplemental Table 2:** RFO levels shown as percent dry weight in a dry bean diversity panel across twelve site-years organized by market class. The ranking of mean RFO content for each genotype is indicated in superscript.

|  | **2021** | | | |  | **2022** | | | |  | **2023** | | | |  | **Genotype** | |
| --- | --- | --- | --- | --- | --- | --- | --- | --- | --- | --- | --- | --- | --- | --- | --- | --- | --- |
| **Genotype** | **Bow Island** | **Fairfield** | **Lethbridge** | **Vauxhall** |  | **Bow Island** | **Fairfield** | **Lethbridge** | **Vauxhall** |  | **Bow Island** | **Fairfield** | **Lethbridge** | **Vauxhall** |  | **Mean** | **Rank** |
| **Pinto** |  |  |  |  |  |  |  |  |  |  |  |  |  |  |  |  |  |
| ‘AAC Expedition’ | 3.14 | 3.31 | 3.26 | 3.64 |  | 3.23 | 3.40 | 3.33 | 3.53 |  | 4.27 | 3.61 | 3.59 | 3.67 |  | 3.50 | 9 |
| ‘AAC Explorer’ | 3.58 | 3.71 | 3.41 | 3.75 |  | 3.28 | 3.61 | 3.52 | 3.74 |  | 4.30 | 3.71 | 3.59 | 4.20 |  | 3.70 | 22 |
| ‘CDC WM-3’ | 3.33 | 3.50 | 3.53 | 3.62 |  | 3.34 | 3.35 | 3.74 | 3.29 |  | 4.24 | 4.04 | 4.42 | 3.76 |  | 3.68 | 21 |
| ‘Island’ | 3.22 | 3.55 | 3.46 | 3.67 |  | 3.46 | 3.67 | 3.65 | 3.66 |  | 4.19 | 4.11 | 3.43 | 3.56 |  | 3.64 | 19 |
| ‘AAC PT600’ | 2.67 | 2.97 | 3.06 | 2.87 |  | 3.40 | 3.02 | 2.99 | 3.54 |  | 3.71 | 3.30 | 3.18 | 3.83 |  | 3.21 | 12 |
| ‘AAC PT601’ | 3.32 | 3.52 | 3.41 | 3.44 |  | 3.00 | 3.29 | 3.00 | 3.48 |  | 3.67 | 3.94 | 3.62 | 3.87 |  | 3.46 | 8 |
| L18PS644 | 3.32 | 2.85 | 2.87 | 3.45 |  | 3.06 | 3.09 | 3.45 | 3.72 |  | 3.88 | 3.44 | 3.44 | 3.68 |  | 3.36 | 5 |
| L19PS653 | 3.22 | 3.56 | 3.21 | 3.41 |  | 3.09 | 3.09 | 2.98 | 3.62 |  | 3.93 | 4.08 | 3.62 | 3.32 |  | 3.43 | 7 |
| L19PS654 | 3.50 | 3.87 | 3.54 | 3.68 |  | 3.72 | 3.56 | 3.14 | 3.67 |  | 4.13 | 3.99 | 3.73 | 3.97 |  | 3.71 | 24 |
|  |  |  |  |  |  |  |  |  |  |  |  |  |  |  |  |  |  |
| **Great Northern** |  |  |  |  |  |  |  |  |  |  |  |  |  |  |  |  |  |
| ‘AAC Whitehorse’ | 3.17 | 3.42 | 3.34 | 3.57 |  | 3.16 | 3.42 | 3.38 | 3.14 |  | 4.39 | 3.70 | 3.55 | 4.04 |  | 3.52 | 10 |
| ‘AAC Whitestar’ | 3.29 | 3.51 | 3.57 | 3.44 |  | 3.38 | 3.64 | 3.40 | 3.44 |  | 4.07 | 3.56 | 3.74 | 3.61 |  | 3.55 | 13 |
| ‘AAC GN963’ | 3.31 | 3.66 | 3.30 | 3.66 |  | 3.53 | 3.58 | 3.34 | 3.45 |  | 4.07 | 3.72 | 3.87 | 4.15 |  | 3.64 | 20 |
| L17GN964 | 3.31 | 3.84 | 3.44 | 3.64 |  | 3.16 | 3.54 | 3.33 | 3.69 |  | 4.29 | 3.60 | 3.57 | 4.07 |  | 3.62 | 18 |
| ‘AAC Alberta North’ | 3.33 | 3.36 | 3.35 | 3.46 |  | 3.61 | 3.40 | 3.43 | 3.40 |  | 4.10 | 3.72 | 3.61 | 4.04 |  | 3.57 | 14 |
| ‘Resolute’ | 3.39 | 3.73 | 3.53 | 3.60 |  | 3.40 | 3.40 | 3.17 | 3.76 |  | 4.59 | 3.78 | 3.28 | 3.84 |  | 3.62 | 17 |
|  |  |  |  |  |  |  |  |  |  |  |  |  |  |  |  |  |  |
| **Yellow** |  |  |  |  |  |  |  |  |  |  |  |  |  |  |  |  |  |
| ‘AAC Y012’ | 2.91 | 3.15 | 2.94 | 3.47 |  | 3.13 | 3.13 | 3.05 | 2.94 |  | 3.93 | 3.54 | 3.55 | 3.33 |  | 3.26 | 3 |
| ‘AAC Y015’ | 3.07 | 3.32 | 3.09 | 3.44 |  | 2.73 | 3.36 | 3.37 | 3.30 |  | 3.90 | 3.58 | 3.28 | 3.82 |  | 3.36 | 6 |
| ‘AAC Y073’ | 2.93 | 3.01 | 3.04 | 3.23 |  | 2.73 | 2.50 | 2.91 | 2.51 |  | 3.78 | 2.96 | 3.42 | 3.30 |  | 3.03 | 1 |
| ‘CDC Sunburst’ | 3.30 | 3.33 | 3.50 | 3.32 |  | 3.32 | 3.39 | 3.17 | 3.16 |  | 4.40 | 3.59 | 3.85 | 4.06 |  | 3.53 | 11 |
| L19YL203 | 3.43 | 3.70 | 3.42 | 3.77 |  | 3.11 | 3.35 | 3.56 | 3.49 |  | 4.25 | 3.69 | 3.62 | 3.79 |  | 3.60 | 16 |
|  |  |  |  |  |  |  |  |  |  |  |  |  |  |  |  |  |  |
| **Black** |  |  |  |  |  |  |  |  |  |  |  |  |  |  |  |  |  |
| ‘AC Black Diamond 2’ | 3.57 | 3.67 | 3.50 | 3.73 |  | 3.44 | 3.69 | 3.41 | 3.79 |  | 4.49 | 3.93 | 3.81 | 3.40 |  | 3.70 | 23 |
| ‘AAC Black Diamond ’ | 3.56 | 3.45 | 3.24 | 3.53 |  | 3.42 | 3.37 | 3.30 | 3.67 |  | 4.39 | 3.73 | 3.93 | 3.32 |  | 3.57 | 15 |
| ‘CDC Blackstrap’ | 4.06 | 4.08 | 3.62 | 4.12 |  | 3.76 | 4.16 | 4.19 | 4.26 |  | 4.83 | 4.27 | 4.52 | 4.55 |  | 4.20 | 24 |
|  |  |  |  |  |  |  |  |  |  |  |  |  |  |  |  |  |  |
| **Cranberry** |  |  |  |  |  |  |  |  |  |  |  |  |  |  |  |  |  |
| ‘AAC Cranford’ | 2.80 | 3.34 | 3.08 | 3.39 |  | 3.20 | 3.08 | 3.27 | 2.91 |  | 4.14 | 3.35 | 3.11 | 3.49 |  | 3.26 | 4 |
|  |  |  |  |  |  |  |  |  |  |  |  |  |  |  |  |  |  |
| **Red** |  |  |  |  |  |  |  |  |  |  |  |  |  |  |  |  |  |
| ‘AC Redbond’ | 3.10 | 3.28 | 2.77 | 3.05 |  | 3.26 | 3.11 | 3.01 | 3.15 |  | 3.67 | 3.24 | 3.08 | 3.63 |  | 3.20 | 2 |
|  |  |  |  |  |  |  |  |  |  |  |  |  |  |  |  |  |  |
| **Environment Mean** | 3.29 | 3.48 | 3.31 | 3.54 |  | 3.28 | 3.38 | 3.34 | 3.47 |  | 4.15 | 3.70 | 3.63 | 3.78 |  |  |  |
| **LSD_0.05_** | 0.13 | 0.21 | 0.16 | 0.09 |  | 0.14 | 0.15 | 0.21 | 0.15 |  | 0.16 | 0.18 | 0.18 | 0.17 |  |  |  |

**Supplemental Table 3:** Sucrose levels shown as percent dry weight in a dry bean diversity panel across twelve site-years organized by market class. The ranking of mean sucrose content for each genotype is indicated in superscript.

|  | **2021** | | | |  | **2022** | | | |  | **2023** | | | |  | **Genotype** | |
| --- | --- | --- | --- | --- | --- | --- | --- | --- | --- | --- | --- | --- | --- | --- | --- | --- | --- |
| **Genotype** | **Bow Island** | **Fairfield** | **Lethbridge** | **Vauxhall** |  | **Bow Island** | **Fairfield** | **Lethbridge** | **Vauxhall** |  | **Bow Island** | **Fairfield** | **Lethbridge** | **Vauxhall** |  | **Mean** | **Rank** |
| **Pinto** |  |  |  |  |  |  |  |  |  |  |  |  |  |  |  |  |  |
| ‘AAC Expedition’ | 2.67 | 2.97 | 3.06 | 2.87 |  | 3.40 | 3.02 | 2.99 | 3.54 |  | 3.71 | 3.30 | 3.18 | 3.83 |  | 3.21 | 17 |
| ‘AAC Explorer’ | 2.54 | 2.66 | 2.67 | 2.58 |  | 2.89 | 2.91 | 2.72 | 3.01 |  | 3.33 | 2.86 | 3.02 | 3.10 |  | 2.86 | 12 |
| ‘CDC WM-3’ | 2.55 | 3.09 | 3.25 | 2.82 |  | 3.20 | 2.97 | 3.02 | 3.39 |  | 3.19 | 3.44 | 3.07 | 3.55 |  | 3.13 | 16 |
| ‘Island’ | 1.74 | 2.30 | 2.09 | 1.98 |  | 2.21 | 1.96 | 2.44 | 2.49 |  | 2.23 | 2.59 | 2.34 | 2.54 |  | 2.24 | 1 |
| ‘AAC PT600’ | 2.85 | 3.14 | 3.45 | 2.81 |  | 3.36 | 3.24 | 3.42 | 3.55 |  | 3.42 | 3.72 | 3.33 | 3.81 |  | 3.34 | 19 |
| ‘AAC PT601’ | 2.94 | 3.04 | 3.55 | 3.05 |  | 3.25 | 3.19 | 3.39 | 4.17 |  | 3.65 | 3.90 | 3.34 | 4.18 |  | 3.47 | 23 |
| L18PS644 | 2.90 | 3.17 | 3.29 | 3.05 |  | 3.72 | 3.40 | 3.59 | 3.87 |  | 3.73 | 3.70 | 3.66 | 3.95 |  | 3.50 | 24 |
| L19PS653 | 2.08 | 2.14 | 2.24 | 2.04 |  | 2.25 | 2.34 | 2.35 | 2.50 |  | 2.77 | 2.60 | 2.39 | 2.72 |  | 2.37 | 3 |
| L19PS654 | 2.88 | 3.11 | 3.15 | 3.12 |  | 3.40 | 3.29 | 3.32 | 3.75 |  | 3.55 | 3.77 | 3.35 | 3.68 |  | 3.36 | 20 |
|  |  |  |  |  |  |  |  |  |  |  |  |  |  |  |  |  |  |
| **Great Northern** |  |  |  |  |  |  |  |  |  |  |  |  |  |  |  |  |  |
| ‘AAC Whitehorse’ | 2.24 | 2.42 | 2.66 | 2.49 |  | 2.46 | 2.43 | 2.54 | 2.86 |  | 2.71 | 2.75 | 2.57 | 2.91 |  | 2.59 | 6 |
| ‘AAC Whitestar’ | 2.28 | 2.83 | 2.71 | 2.48 |  | 2.77 | 2.59 | 3.11 | 3.10 |  | 2.97 | 2.75 | 2.73 | 2.97 |  | 2.78 | 9 |
| ‘AAC GN963’ | 2.39 | 2.71 | 2.63 | 2.38 |  | 2.32 | 2.36 | 2.56 | 2.73 |  | 2.68 | 2.67 | 2.52 | 2.97 |  | 2.58 | 5 |
| L17GN964 | 2.28 | 2.42 | 2.72 | 2.27 |  | 2.44 | 2.56 | 2.45 | 2.63 |  | 2.77 | 2.66 | 2.42 | 2.95 |  | 2.55 | 4 |
| ‘AAC Alberta North’ | 2.26 | 2.51 | 2.65 | 2.49 |  | 2.65 | 2.56 | 2.74 | 2.66 |  | 2.88 | 2.73 | 2.47 | 2.97 |  | 2.63 | 7 |
| ‘Resolute’ | 2.81 | 2.83 | 3.13 | 2.59 |  | 3.02 | 2.95 | 3.33 | 3.16 |  | 2.78 | 3.08 | 2.95 | 3.24 |  | 2.99 | 13 |
|  |  |  |  |  |  |  |  |  |  |  |  |  |  |  |  |  |  |
| **Yellow** |  |  |  |  |  |  |  |  |  |  |  |  |  |  |  |  |  |
| ‘AAC Y012’ | 2.77 | 3.44 | 3.07 | 3.30 |  | 3.16 | 3.38 | 2.79 | 3.35 |  | 3.17 | 3.50 | 3.29 | 3.92 |  | 3.26 | 18 |
| ‘AAC Y015’ | 3.20 | 3.49 | 3.12 | 3.41 |  | 3.50 | 3.41 | 3.28 | 3.49 |  | 3.29 | 3.48 | 3.42 | 3.57 |  | 3.39 | 22 |
| ‘AAC Y073’ | 2.92 | 3.24 | 3.12 | 3.25 |  | 3.41 | 3.40 | 3.57 | 3.64 |  | 3.47 | 3.52 | 3.32 | 3.62 |  | 3.37 | 21 |
| ‘CDC Sunburst’ | 2.21 | 3.00 | 2.82 | 2.31 |  | 2.67 | 2.81 | 2.62 | 2.89 |  | 2.70 | 2.90 | 2.84 | 3.05 |  | 2.74 | 8 |
| L19YL203 | 3.31 | 3.88 | 3.27 | 3.47 |  | 3.63 | 3.57 | 3.16 | 3.74 |  | 3.46 | 3.61 | 3.60 | 3.91 |  | 3.55 | 25 |
|  |  |  |  |  |  |  |  |  |  |  |  |  |  |  |  |  |  |
| **Black** |  |  |  |  |  |  |  |  |  |  |  |  |  |  |  |  |  |
| ‘AC Black Diamond 2’ | 2.51 | 2.87 | 2.68 | 2.51 |  | 2.72 | 3.26 | 3.16 | 3.73 |  | 2.88 | 3.09 | 2.91 | 3.83 |  | 3.01 | 14 |
| ‘AAC Black Diamond ’ | 2.63 | 2.61 | 2.84 | 2.85 |  | 2.85 | 3.21 | 3.12 | 3.73 |  | 3.24 | 3.23 | 3.25 | 3.59 |  | 3.10 | 15 |
| ‘CDC Blackstrap’ | 1.99 | 2.13 | 1.96 | 1.91 |  | 2.15 | 2.58 | 2.30 | 2.66 |  | 2.18 | 2.15 | 2.52 | 2.69 |  | 2.27 | 2 |
|  |  |  |  |  |  |  |  |  |  |  |  |  |  |  |  |  |  |
| **Cranberry** |  |  |  |  |  |  |  |  |  |  |  |  |  |  |  |  |  |
| ‘AAC Cranford’ | 2.69 | 2.88 | 2.74 | 2.54 |  | 2.35 | 2.96 | 3.10 | 3.06 |  | 2.85 | 2.73 | 2.69 | 3.27 |  | 2.82 | 11 |
|  |  |  |  |  |  |  |  |  |  |  |  |  |  |  |  |  |  |
| **Red** |  |  |  |  |  |  |  |  |  |  |  |  |  |  |  |  |  |
| ‘AC Redbond’ | 2.67 | 2.55 | 2.43 | 2.52 |  | 2.79 | 2.83 | 3.09 | 2.94 |  | 3.26 | 2.97 | 2.73 | 2.92 |  | 2.81 | 10 |
|  |  |  |  |  |  |  |  |  |  |  |  |  |  |  |  |  |  |
| **Environment Mean** | 3.29 | 3.48 | 3.31 | 3.54 |  | 3.28 | 3.38 | 3.34 | 3.47 |  | 4.15 | 3.70 | 3.63 | 3.78 |  |  |  |
| **LSD_0.05_** | 0.13 | 0.21 | 0.16 | 0.09 |  | 0.14 | 0.15 | 0.21 | 0.15 |  | 0.16 | 0.18 | 0.18 | 0.17 |  |  |  |

**Supplemental Table 4:** Starch levels shown as percent dry weight in a dry bean diversity panel across twelve site-years organized by market class. The ranking of mean starch content for each genotype is indicated in superscript.

|  | **2021** | | | |  | **2022** | | | |  | **2023** | | | |  | **Genotype** | |
| --- | --- | --- | --- | --- | --- | --- | --- | --- | --- | --- | --- | --- | --- | --- | --- | --- | --- |
| **Genotype** | **Bow Island** | **Fairfield** | **Lethbridge** | **Vauxhall** |  | **Bow Island** | **Fairfield** | **Lethbridge** | **Vauxhall** |  | **Bow Island** | **Fairfield** | **Lethbridge** | **Vauxhall** |  | **Mean** | **Rank** |
| **Pinto** |  |  |  |  |  |  |  |  |  |  |  |  |  |  |  |  |  |
| ‘AAC Expedition’ | 37.2 | 37.3 | 37.9 | 35.9 |  | 35.9 | 38.9 | 37.7 | 34.6 |  | 37.2 | 37.7 | 38.0 | 40.2 |  | 37.4 | 16 |
| ‘AAC Explorer’ | 36.9 | 36.1 | 39.2 | 36.2 |  | 37.5 | 38.1 | 36.7 | 35.0 |  | 37.0 | 36.6 | 30.0 | 37.6 |  | 36.4 | 14 |
| ‘CDC WM-3’ | 35.2 | 34.7 | 36.6 | 36.5 |  | 34.1 | 37.4 | 36.8 | 30.1 |  | 38.1 | 37.0 | 34.1 | 36.0 |  | 35.6 | 5 |
| ‘Island’ | 39.1 | 39.9 | 39.3 | 37.9 |  | 38.0 | 42.0 | 40.1 | 39.4 |  | 40.1 | 40.3 | 40.2 | 39.1 |  | 39.6 | 25 |
| ‘AAC PT600’ | 39.8 | 37.9 | 42.9 | 37.7 |  | 32.5 | 41.9 | 38.9 | 36.2 |  | 41.0 | 41.5 | 39.7 | 38.6 |  | 39.1 | 23 |
| ‘AAC PT601’ | 40.4 | 38.0 | 41.9 | 37.4 |  | 29.6 | 40.8 | 38.6 | 38.8 |  | 39.5 | 39.9 | 44.4 | 38.8 |  | 39.0 | 22 |
| L18PS644 | 39.2 | 39.8 | 40.9 | 37.1 |  | 38.1 | 40.9 | 40.3 | 34.4 |  | 39.4 | 41.0 | 38.7 | 40.4 |  | 39.2 | 24 |
| L19PS653 | 37.7 | 38.4 | 40.1 | 38.2 |  | 35.2 | 40.8 | 38.3 | 39.8 |  | 40.1 | 39.8 | 40.5 | 39.3 |  | 39.0 | 21 |
| L19PS654 | 38.4 | 36.3 | 40.6 | 36.7 |  | 33.4 | 40.5 | 38.0 | 36.3 |  | 39.1 | 38.7 | 39.2 | 35.9 |  | 37.8 | 19 |
|  |  |  |  |  |  |  |  |  |  |  |  |  |  |  |  |  |  |
| **Great Northern** |  |  |  |  |  |  |  |  |  |  |  |  |  |  |  |  |  |
| ‘AAC Whitehorse’ | 38.4 | 39.4 | 41.8 | 35.9 |  | 30.1 | 39.2 | 36.8 | 38.8 |  | 37.5 | 36.5 | 38.2 | 37.9 |  | 37.5 | 18 |
| ‘AAC Whitestar’ | 36.7 | 37.7 | 39.6 | 35.9 |  | 30.2 | 36.8 | 35.5 | 33.7 |  | 36.7 | 35.9 | 36.2 | 37.4 |  | 36.0 | 12 |
| ‘AAC GN963’ | 38.6 | 37.2 | 42.4 | 36.1 |  | 39.7 | 37.4 | 36.9 | 36.1 |  | 39.4 | 39.0 | 36.0 | 38.7 |  | 38.1 | 20 |
| L17GN964 | 38.7 | 38.5 | 39.7 | 35.9 |  | 30.2 | 38.9 | 40.0 | 36.5 |  | 38.8 | 38.3 | 36.5 | 38.0 |  | 37.5 | 17 |
| ‘AAC Alberta North’ | 39.0 | 37.4 | 36.4 | 36.7 |  | 33.0 | 38.7 | 37.1 | 32.3 |  | 38.6 | 36.3 | 37.2 | 38.5 |  | 36.8 | 15 |
| ‘Resolute’ | 38.7 | 36.3 | 37.9 | 36.1 |  | 30.1 | 35.7 | 35.1 | 38.7 |  | 37.5 | 37.8 | 31.9 | 35.3 |  | 35.9 | 9 |
|  |  |  |  |  |  |  |  |  |  |  |  |  |  |  |  |  |  |
| **Yellow** |  |  |  |  |  |  |  |  |  |  |  |  |  |  |  |  |  |
| ‘AAC Y012’ | 36.8 | 34.5 | 37.8 | 35.4 |  | 32.6 | 38.2 | 36.9 | 29.6 |  | 37.6 | 37.2 | 35.4 | 37.2 |  | 35.8 | 7 |
| ‘AAC Y015’ | 36.2 | 36.6 | 37.5 | 34.6 |  | 34.6 | 37.9 | 36.6 | 30.7 |  | 36.6 | 38.6 | 36.0 | 35.7 |  | 36.0 | 11 |
| ‘AAC Y073’ | 36.5 | 36.9 | 36.2 | 35.4 |  | 33.4 | 36.2 | 37.2 | 33.1 |  | 36.7 | 37.1 | 35.7 | 33.4 |  | 35.7 | 6 |
| ‘CDC Sunburst’ | 36.3 | 35.7 | 40.3 | 35.8 |  | 32.0 | 34.8 | 32.0 | 35.6 |  | 38.5 | 32.7 | 32.5 | 32.5 |  | 34.9 | 3 |
| L19YL203 | 35.7 | 37.0 | 38.9 | 34.9 |  | 33.5 | 36.3 | 37.3 | 36.0 |  | 36.9 | 35.3 | 33.9 | 35.8 |  | 36.0 | 10 |
|  |  |  |  |  |  |  |  |  |  |  |  |  |  |  |  |  |  |
| **Black** |  |  |  |  |  |  |  |  |  |  |  |  |  |  |  |  |  |
| ‘AC Black Diamond 2’ | 36.1 | 36.2 | 34.3 | 34.7 |  | 22.3 | 34.1 | 33.5 | 31.8 |  | 34.9 | 35.5 | 35.1 | 35.1 |  | 33.6 | 1 |
| ‘AAC Black Diamond ’ | 37.2 | 32.9 | 38.1 | 34.2 |  | 34.9 | 37.9 | 35.1 | 34.9 |  | 36.8 | 36.7 | 37.1 | 37.0 |  | 36.1 | 13 |
| ‘CDC Blackstrap’ | 33.7 | 34.1 | 36.0 | 32.3 |  | 25.8 | 36.5 | 34.3 | 33.0 |  | 34.6 | 35.5 | 34.5 | 35.6 |  | 33.8 | 2 |
|  |  |  |  |  |  |  |  |  |  |  |  |  |  |  |  |  |  |
| **Cranberry** |  |  |  |  |  |  |  |  |  |  |  |  |  |  |  |  |  |
| ‘AAC Cranford’ | 37.9 | 36.8 | 37.8 | 33.9 |  | 35.3 | 37.6 | 37.5 | 36.1 |  | 37.2 | 31.8 | 31.6 | 32.8 |  | 35.5 | 4 |
|  |  |  |  |  |  |  |  |  |  |  |  |  |  |  |  |  |  |
| **Red** |  |  |  |  |  |  |  |  |  |  |  |  |  |  |  |  |  |
| ‘AC Redbond’ | 36.9 | 36.5 | 36.2 | 35.2 |  | 35.1 | 35.4 | 35.0 | 36.5 |  | 37.0 | 36.4 | 36.0 | 33.5 |  | 35.8 | 8 |
|  |  |  |  |  |  |  |  |  |  |  |  |  |  |  |  |  |  |
| **Environment Mean** | 3.29 | 3.48 | 3.31 | 3.54 |  | 3.28 | 3.38 | 3.34 | 3.47 |  | 4.15 | 3.70 | 3.63 | 3.78 |  |  |  |
| **LSD_0.05_** | 0.13 | 0.21 | 0.16 | 0.09 |  | 0.14 | 0.15 | 0.21 | 0.15 |  | 0.16 | 0.18 | 0.18 | 0.17 |  |  |  |

**Supplemental Table 5**: Intra-genotype correlations among nutrient classes across all treatments during the heat stress experiment. Bold indicates a statistically significant correlation (*p*<0.05, n=18)

| **Genotype** | Correlation Coefficient | | | | | |
| --- | --- | --- | --- | --- | --- | --- |
|  | RFO:Sucrose | Sucrose:Protein | Sucrose:Starch | RFO:Protein | RFO:Starch | Protein:Starch |
| **Pinto** |  |  |  |  |  |  |
| ‘AAC Expedition’ | 0.20 | **-0.63** | 0.38 | -0.16 | **0.49** | **-0.59** |
| ‘AAC Explorer’ | 0.18 | **-0.70** | 0.42 | -0.18 | 0.34 | **-0.74** |
| ‘CDC WM3’ | -0.06 | **-0.68** | **0.52** | 0.04 | -0.15 | -0.42 |
| ‘Island’ | -0.07 | -0.16 | 0.37 | **-0.48** | 0.15 | **-0.82** |
| L18PS644 | **0.48** | -0.29 | 0.46 | 0.04 | -0.24 | -0.41 |
| L19PS654 | -0.34 | -0.45 | 0.40 | 0.02 | 0.09 | **-0.58** |
|  |  |  |  |  |  |  |
| **Yellow** |  |  |  |  |  |  |
| ‘AAC Y012’ | 0.27 | -0.39 | 0.33 | 0.19 | 0.14 | **-0.65** |
| ‘AAC Y015’ | 0.24 | -0.31 | 0.21 | -0.13 | 0.07 | -0.28 |
| ‘AAC Y073’ | 0.13 | -0.61 | **0.52** | -0.16 | 0.19 | **-0.52** |
| ‘CDC Sunburst’ | -0.18 | 0.01 | **-0.60** | **0.51** | 0.00 | -0.35 |
| L19YL203 | **0.59** | -0.39 | 0.41 | -0.19 | 0.03 | -0.21 |
|  |  |  |  |  |  |  |
| **Cranberry** |  |  |  |  |  |  |
| ‘AAC Cranford’ | **-0.52** | -0.44 | -0.26 | -0.08 | **0.50** | -0.41 |
|  |  |  |  |  |  |  |
| **Mean** | 0.08 | -0.42 | 0.26 | -0.05 | 0.14 | **-0.50** |
